# Fenofibrate Reduces the Asthma-Related Fibroblast-To-Myofibroblast Transition by TGF-Β/Smad2/3 Signaling Attenuation and Connexin 43-Dependent Phenotype Destabilization

**DOI:** 10.3390/ijms19092571

**Published:** 2018-08-29

**Authors:** Milena Paw, Dawid Wnuk, Dominika Kądziołka, Aleksandra Sęk, Sławomir Lasota, Jarosław Czyż, Zbigniew Madeja, Marta Michalik

**Affiliations:** 1 Faculty of Biophysics, Biochemistry and Biotechnology, Department of Cell Biology, Jagiellonian University, Gronostajowa 7, 30-378 Kraków, Poland; milena.paw@uj.edu.pl (M.P.); dawid.wnuk@doctoral.uj.edu.pl (D.W.); doominnika@o2.pl (D.K.); aleksandra.sek@interia.pl (A.S.); slawomir.lasota@uj.edu.pl (S.L.); jarek.czyz@uj.edu.pl (J.C.); z.madeja@uj.edu.pl (Z.M.); 2 Nencki Institute of Experimental Biology, Laboratory of Intracellular Ion Channels, 02-093 Warsaw, Poland

**Keywords:** fibroblast-to-myofibroblast transition, TGF-β/Smad signaling, fenofibrate, bronchial asthma, actin cytoskeleton architecture, connexin 43

## Abstract

The activation of human bronchial fibroblasts by transforming growth factor-β_1_ (TGF-β_1_) leads to the formation of highly contractile myofibroblasts in the process of the fibroblast–myofibroblast transition (FMT). This process is crucial for subepithelial fibrosis and bronchial wall remodeling in asthma. However, this process evades current therapeutic asthma treatment strategies. Since our previous studies showed the attenuation of the TGF-β_1_-induced FMT in response to lipid-lowering agents (e.g., statins), we were interested to see whether a corresponding effect could be obtained upon administration of hypolipidemic agents. In this study, we investigated the effect of fenofibrate on FMT efficiency in populations of bronchial fibroblasts derived from asthmatic patients. Fenofibrate exerted a dose-dependent inhibitory effect on the FMT, even though it did not efficiently affect the expression of α-smooth muscle actin (α-SMA; marker of myofibroblasts); however, it considerably reduced its incorporation into stress fibers through connexin 43 regulation. This effect was accompanied by disturbances in the actin cytoskeleton architecture, impairments in the maturation of focal adhesions, and the fenofibrate-induced deactivation of TGF-β_1_/Smad2/3 signaling. These data suggest that fenofibrate interferes with myofibroblastic differentiation during asthma-related subepithelial fibrosis. The data indicate the potential application of fenofibrate in the therapy and prevention of bronchial remodeling during the asthmatic process.

## 1. Introduction

Contemporary views on asthma pathophysiology suggest that the asthmatic process in proximal bronchi is associated with chronic inflammation-induced bronchial wall remodeling [1,2]. Bronchial asthma is manifested by a wide range of histopathological changes in the bronchial wall. These changes comprise epithelial dysfunction and an unbalanced increase in the airway smooth muscle mass and subepithelial fibrosis, which is associated with the abnormal activation of bronchial fibroblasts [3]. Because of the bronchial wall remodeling, a functional impairment of asthmatic bronchi and chronic airflow obstruction occurs. This impairment is accompanied by chronic inflammation that further promotes pro-fibrotic changes in the bronchial airway walls [4]. In particular, the asthmatic bronchi microenvironment is rich in locally secreted pro-inflammatory cytokines and growth factors (especially TGF-β_1_), which favor the myofibroblastic differentiation of bronchial fibroblasts.

The Global Strategy for Asthma Management and Prevention report clearly shows an increasing number of bronchial asthma cases in the last several decades [5]. Therefore, new therapeutic strategies that would help interrupt this vicious circle are necessary. Whereas inflammation remains the primary target of asthma therapies [6,7], these therapies have a negligible effect on structure and hardly attenuate the structural changes in asthmatic airways [8,9]. This problem underlies the need for elaboration of new therapeutic approaches that directly target the pro-fibrotic processes, including the fibroblast–myofibroblast transition (FMT).

It is generally accepted that the progression of subepithelial fibrosis in asthma is predominantly determined by an increased myofibroblastic differentiation and increased longevity of myofibroblasts, leading to their accumulation in bronchial walls [4,10]. Two basic features of these cells, i.e., their contractility and pro-fibrotic activity, are determined by a well-developed α-smooth-muscle-actin-positive (α-SMA^+^) contractile apparatus and secretion of extracellular matrix (ECM) components, respectively [11,12]. These features are acquired in the FMT process in response to mechanical and/or humoral stimuli, which concomitantly prompt the formation of α-smooth muscle actin (α-SMA) microfilaments in phenotypically plastic fibroblasts [13,14]. Due to the crucial role of the FMT in subepithelial fibrosis and the involvement of subepithelial fibrosis in the asthmatic process, it is conceivable that pharmacologic attenuation of this process may constitute a milestone in asthma treatment. An increasing amount of evidence shows that lipid metabolism disorders that lead to obesity are significant risk factors that favor asthma progression [15,16]. Our previous study indicated the impact of statins (lovastatin and squalestatin) on the progression of subepithelial fibrosis during asthma, which was accomplished through the attenuation of phenotypic shifts of bronchial fibroblasts into myofibroblasts [17]. These data attracted our attention for the potential application of other anti-hyperlipidemic drugs in asthma treatment.

Numerous reports described an anti-inflammatory and anti-fibrotic potential of fenofibrate. Fenofibrate is a Food and Drug Administration (FDA)-approved agent, routinely used in hyperlipidemia treatment [18]. Studies performed with human, rat, and mouse models of lung disorders, including asthma and idiopathic pulmonary fibrosis [19,20,21,22], suggest that fenofibrate can be useful in the elaboration of pharmacotherapeutic approaches targeting fibrotic changes in the subepithelial layer of asthmatic bronchi. However, the effect of fenofibrate on bronchial wall remodeling and FMT efficiency in asthma is yet to be studied. To fill this gap, we estimated the effect of fenofibrate on the TGF-β_1_-induced FMT in populations of bronchial fibroblasts. For this purpose, we used an experimental model based on human bronchial fibroblasts propagated in vitro from the ex vivo bronchial biopsies of patients with diagnosed asthma.

## 2. Results

### 2.1. Fenofibrate Attenuates the TGF-Β_1_-Induced Differentiation of Human Bronchial Fibroblasts into Myofibroblasts

**Figure 1:**
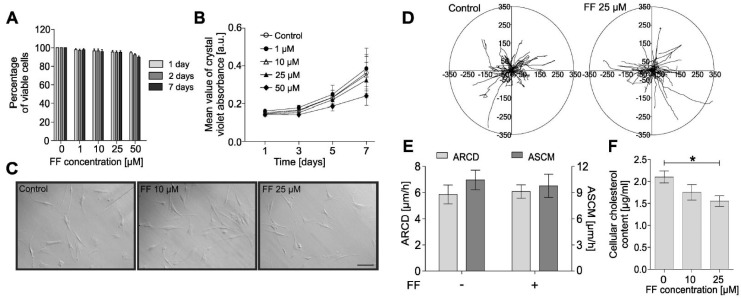
Fenofibrate does not affect the viability, proliferation, morphology, or motility of human bronchial fibroblasts (HBFs). (**A**) HBFs derived from asthma patients were cultured in the absence or presence of fenofibrate (FF; 1–50 µM) for one, two, and seven days, and the number of viable cells was detected using the fluorescein diacetate/ethidium bromide (FDA/EtBr) test. (**B**) Proliferation of HBFs after FF (0–50 µM) administration was measured after one, three, five, and seven days of cell cultivation, presented as a mean value of crystal violet absorbance. (**C**) Representative images of HBF morphologies after treatment with FF for 48 h (integrated modulation contrast, IMC). Scale bar = 25 µm. (**D**,**E**) HBFs were cultured in the absence or presence of FF (25 μM) for 48 h. The results of time-lapse monitoring of HBF movement are presented as circular diagrams, with the starting point of each trajectory situated at the plot center and column charts summarizing the effect of FF on the average speed of cell movement (ASCM; µm/h) and the average rate of cell displacement (ARCD; µm) parameters. (**F**) Intracellular cholesterol levels of HBF populations (*n* = 10) were measured with an Amplex Cholesterol Assay Kit after seven days of FF treatment (10 or 25 µM) in three independent experiments and are shown on a graph. Data are the mean ± standard error of the mean (SEM) of six independent experiments. Statistical significances were tested using one-way (**A**,**C**,**F**) or two-way ANOVA (**B**) with the Bonferroni multiple comparison post hoc test; * *p* ≤ 0.05.

Although fenofibrate is commonly used in the therapy of lipid metabolism disorders, it was also reported that fenofibrate inhibits collagen production during lung fibrotic disease [22]. In asthmatic bronchi, collagen is predominantly deposited by bronchial fibroblasts and myofibroblasts; therefore, we estimated the effect of fenofibrate on myofibroblastic differentiation of HBFs derived from asthmatics. Viability tests demonstrated the lack of any cytotoxic effects of fenofibrate on human bronchial fibroblasts (HBFs) at concentrations up to 50 μM (Figure 1A).

Absence of the deleterious effects of fenofibrate on HBFs was further verified by proliferation tests (Figure 1B), followed by morphological analyses (Figure 1C) and time-lapse analyses of HBF motility (Figure 1D,E). These analyses did not reveal any significant effect of fenofibrate on these parameters. However, we observed hypolipemic effects manifested by the analyses of intracellular cholesterol levels, which were significantly reduced in HBFs exposed to 25 µM fenofibrate (Figure 1F).

**Figure 2:**
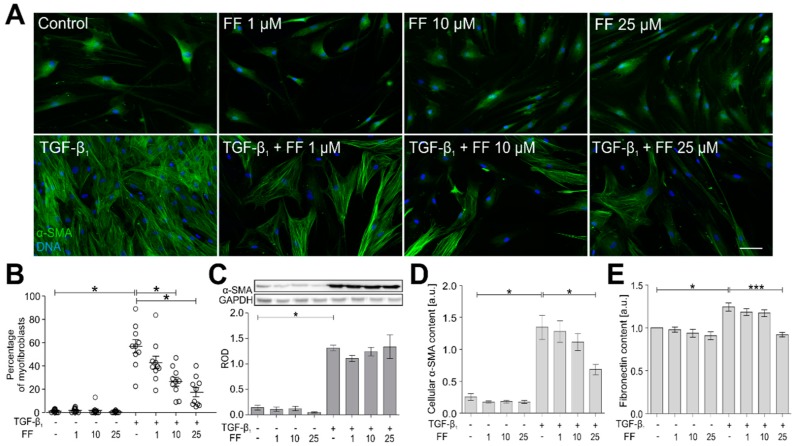
Fenofibrate attenuates the TGF-β_1_-induced phenotypic transition of HBFs into myofibroblasts. (**A**) HBFs were cultured in control conditions or in Dulbecco’s modified Eagle medium (DMEM) supplemented with TGF-β_1_ (5 ng/mL) in the absence or presence of FF (1–25 μM) for seven days. Then, the cells were fixed with 3.7% formaldehyde, permeabilized, and immunostained for α-smooth muscle actin (α-SMA; green) and DNA (blue) as shown on representative images. Scale bar = 25 µm. (**B**) The fraction of cells with prominent α-SMA^+^ stress fibers in HBF populations (*n* = 10) was determined using fluorescence microscopy in three independent experiments. (**C**) Analyses of α-SMA content were carried out in total cell lysates from HBFs cultured as in (**A**) using immunoblotting. Human glyceraldehyde 3-phosphate dehydrogenase (GAPDH) was used as a loading control. The effect of fenofibrate on the α-SMA levels in the TGF-β_1_-treated HBFs is presented as a bar graph and shows densitometric quantification of Western blots. Data are the mean ± SEM of five independent experiments in triplicate. (**D**,**E**) α-SMA and fibronectin contents, respectively, were defined using in-cell ELISA, and the results are presented as the mean value of absorbance (450 nm) reflecting the protein content. Data represent the mean ± SEM carried out on HBFs (*n* = 10), each in triplicate. Statistical significances were tested using one-way ANOVA with the Bonferroni multiple comparison post hoc test; * *p* ≤ 0.05, *** *p* ≤ 0.001. Note that fenofibrate significantly inhibits the formation of α-SMA^+^ stress fibers in the TGF-β_1_-treated HBFs in a dose-dependent manner, and concomitantly, has a slight impact on the total α-SMA level.

Further analyses were performed to estimate the effect of fenofibrate on the TGF-β_1_-induced FMT in HBF populations (*n* = 10). For this purpose, fibroblasts were cultivated for seven days in the presence of fenofibrate (1–50 μM) and in the presence/absence of TGF-β_1_. In response to TGF-β_1_, HBFs efficiently differentiated into highly contractile myofibroblasts, characterized by abundant α-SMA^+^ stress fibers (up to ca. 60% of population; Figure 2A,B).

Fenofibrate reduced this effect in a dose-dependent manner. Interestingly, immunoblot analyses did not reveal any significant impact of fenofibrate on α-SMA levels in the HBF populations (Figure 2C). This somewhat unexpected observation prompted us to verify these data with in-cell ELISA (Figure 2D). These immunoenzymatic studies showed a slight impact of fenofibrate on α-SMA levels, which was less pronounced than the effect on FMT efficiency. Also, decreased content of fibronectin (another marker of myofibroblasts) confirmed the attenuation of phenotypic shifts in TGF-β_1_/fenofibrate-treated HBFs. These observations demonstrate that fenofibrate exerts an inhibitory effect on the TGF-β_1_-induced FMT in HBF populations predominantly via inhibition of α-SMA incorporation into stress fibers.

### 2.2. Actin Cytoskeleton Architecture Is Reorganized by Fenofibrate in TGF-β_1_-Treated HBFs

**Figure 3:**
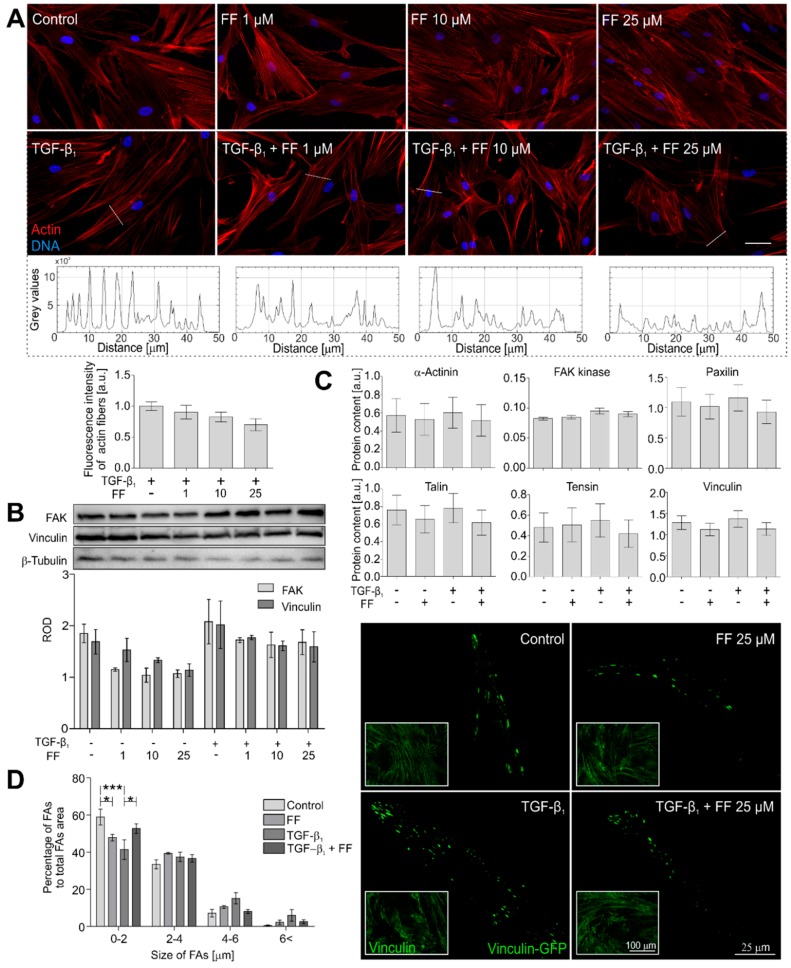
Fenofibrate affects actin cytoskeleton architecture. (**A**) HBFs were cultured in DMEM supplemented with TGF-β_1_ (5 ng/mL) in the absence or presence of FF (1–25 μM) for seven days. Then, the cells were fixed with 3.7% formaldehyde, permeabilized, and immunostained for actin (red) and DNA (blue). Representative images were selected and presented. Intensity of actin fiber fluorescence in sections is presented on plot profiles and quantified in graphs. Scale bar = 25 µm. (**B**) Cellular content of the selected focal adhesion proteins was measured by Western blots and (**C**) in-cell ELISA. Representative images of vinculin-rich focal adhesions are presented, (**D**) quantified and grouped by size. Data represent the mean ± SEM of five independent experiments in all analyses. Statistical significances were tested using one-way ANOVA with the Bonferroni multiple comparison post hoc test; * *p* ≤ 0.05; *** *p* ≤ 0.001. Note that fenofibrate affects the architecture of actin cytoskeletons and arrangement of focal adhesion maturation in the TGF-β_1_-treated HBFs.

To mechanically address the inhibition of α-SMA incorporation into stress fibers, we further concentrated on their actin cytoskeleton architecture in the fenofibrate-treated HBFs. Fenofibrate considerably reduced the thickness and affected cytoplasmic localization of stress fibers in the TGF-β_1_-treated HBFs (Figure 3A).

These microscopic observations were confirmed by fluorometric analyses, which revealed fewer prominent stress fibers in the TGF-β_1_/fenofibrate-treated HBFs. Accordingly, the visualization of vinculin-rich focal adhesions (FAs) in these cells by total internal reflection fluorescence (TIRF) microscopy revealed the reduction in the number of supermature FAs (length > 4 μm) in cells co-treated with TGF-β_1_ and fenofibrate (Figure 3B). However, this effect was not associated with the total number of focal adhesions. However, immunoenzymatic immunoblot analyses and in-cell ELISA showed rather negligible effects of fenofibrate on the expression of FA proteins (Figure 3C,D). These data indicate that fenofibrate exerts an inhibitory effect on the TGF-β_1_-induced incorporation of α-SMA into stress fibers via an effect on actin cytoskeleton architecture.

### 2.3. Fenofibrate Inhibits the TGF-β_1_/Smad Signaling Pathway in TGF-β_1_-Treated HBFs

**Figure 4:**
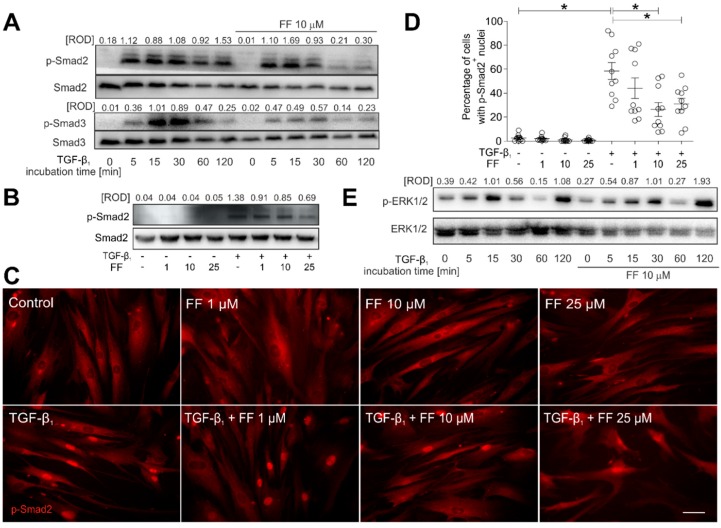
Fenofibrate affects TGF-β_1_-dependent Smad signaling pathway activity in HBFs. (**A**) Cells cultivated in DMEM supplemented with TGF-β1 (5 ng/mL) in the absence or presence of FF (10 μM) for different time points (0–120 min). Then, the cells were lysed, and the total cell lysates were analyzed using immunoblotting. Smad2, Smad3, and their phosphorylated forms were detected using primary antibodies against Smad2, Smad3, phosphorylated (p)-Smad2(Ser465/467) and p-Smad3(Ser423/425), respectively (see Materials and methods; Section 4.7). Representative membranes are shown. Densitometric quantification is presented as values of relative optical densities (ROD) (*n* = 5) of phospho-Smads in relation to Smads (as control proteins). (**B**) Immunoblots with relative optical density (ROD) of p-Smad2(Ser467) in relation to Smad2 are presented in HBFs treated by TGF-β1 (5 ng/mL) in the absence or presence of FF (1–25 μM) for 1 h. The results represent mean ± SEM of five independent experiments. (**C**,**D**) HBFs (*n* = 10) cultured in the conditions described in (**B**) were fixed with 3.7% formaldehyde/PBS, permeabilized, and immunostained for p-Smad2. The fractions of cells with pSmad2^+^ nuclei were determined using fluorescence microscopy. Representative photos were selected. Scale bar = 25 µm. Data represent the mean ± SEM of ten independent experiments in triplicate. (**E**) HBFs cultured in the conditions described in (**A**) were lysed and analyzed by Western blot. Representative immunoblots with relative optical density of p-ERK1/2 in relation to ERK1/2 are presented. Statistical significances were tested using one-way ANOVA with the Bonferroni multiple comparison post hoc Test; * *p* ≤ 0.05. Note that fenofibrate efficiently attenuates the TGF-β_1_-induced Smad signaling, but not the ERK-dependent pathway.

The TGF-β_1_-induced FMT of HBFs is predominantly regulated by the canonical Smad2/Smad3-dependent signaling pathway [17,23,24,25,26]. To elucidate the molecular mechanisms underlying the inhibitory effect of fenofibrate on the TGF-β_1_-induced FMT, we followed the fate of Smad2 and Smad3 in the HBFs that underwent fenofibrate treatment. A significant increase in the phosphorylated (p)-Smad2 and p-Smad3 levels was observed in HBFs after TGF-β_1_ treatment (Figure 4A).

However, Smad2 and Smad3 phosphorylation levels were considerably reduced by fenofibrate in a time- (Figure 4A) and dose-dependent manner (Figure 4B). This effect was correlated with the reduced number of p-Smad2^+^ HBF nuclei observed in the presence of TGF-β_1_/fenofibrate in comparison to the TGF-β_1_ control (from ca. 60% in TGF-β_1_-treated HBFs to 25–30% in TGF-β_1_/fenofibrate-treated HBFs (Figure 4C,D). The discrepancy between the fenofibrate-induced inhibition of TGF-β_1_/Smad signaling and FMT efficiency, as well as a relatively weak effect of fenofibrate on α-SMA expression, prompted us to estimate the involvement of non-canonical signaling pathways in HBF reactions to fenofibrate. Immunoblotting studies did not reveal any effect of fenofibrate on the TGF-β_1_-induced activation of Akt (also known as protein kinase B or PKB) and p38 mitogen-activated protein kinase (also called Cytokinin Specific Binding Protein or CSBP), indicating that neither pathways is involved in the observed phenomena (data not shown). Cyclic changes in extracellular signal–regulated kinases (ERK)1/2 phosphorylation status observed in HBFs upon TGF-β_1_ treatment were not affected by fenofibrate (Figure 4E). ERK1/2 signaling is involved in α-SMA regulation [27,28]. Therefore, these data explain the lack of significant inhibition of the α-SMA levels that accompanies the fenofibrate-induced inhibition of TGF-β_1_/Smad signaling and of α-SMA incorporation into stress fibers in fenofibrate-treated HBF populations.

### 2.4. Connexin 43 Is Involved in Fenofibrate-Induced Attenuation of the FMT in TGF-β_1_-Treated HBFs

**Figure 5:**
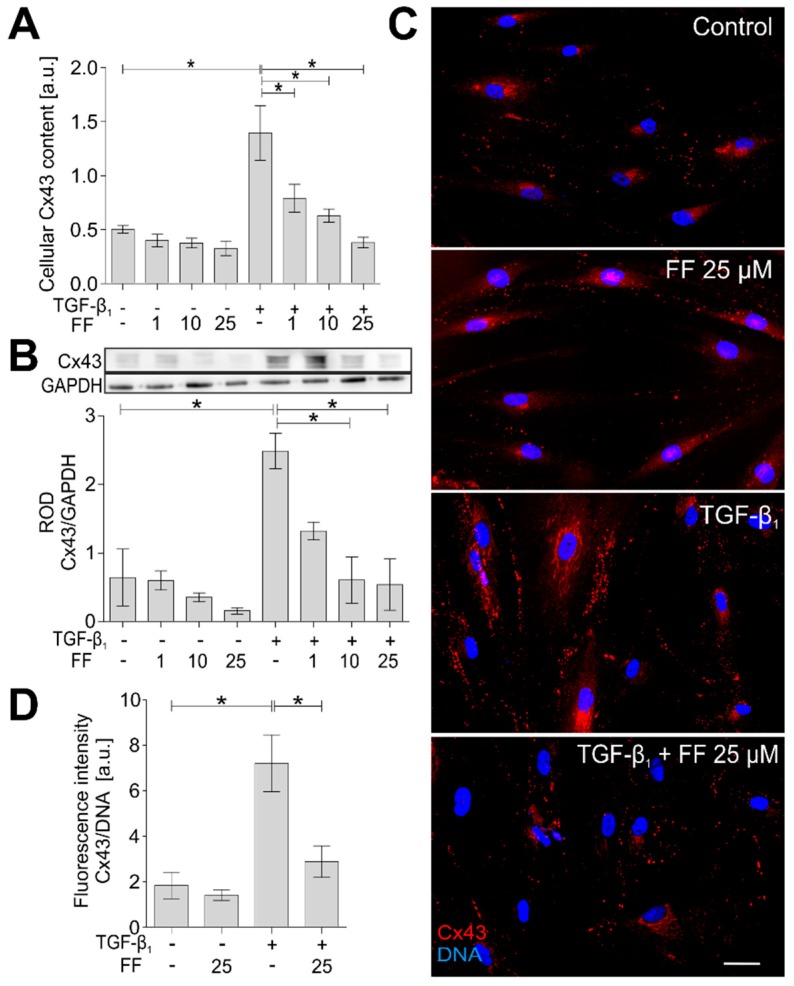
Connexin 43 (Cx43) levels are reduced in TGF-β_1_-treated HBFs in response to fenofibrate administration. (**A**) HBFs were cultured in DMEM supplemented with TGF-β_1_ (5 ng/mL) in the absence or presence of FF (25 μM) for seven days. Cellular content of Cx43 was measured by in-cell ELISA and (**B**) Western blot analyses of total cellular lysates. Representative immunoblots are presented; bar graphs show the relative optical density of densitometrically quantified Cx43 protein levels in relation to GAPDH as a housekeeping protein. (**C**) HBFs were cultured in DMEM supplemented with TGF-β_1_ (5 ng/mL) in the presence or absence of FF (25 μM) for seven days and immunostained for Cx43 (red) and DNA (blue). Representative images are presented. Scale bar = 25 µm. (**D**) Levels of Cx43 were identified using fluorimetry analyses and presented in relation to the control signal of DNA. Data represent the mean ± SEM of five independent experiments in all analyses. Statistical significances were tested using one-way ANOVA with the Bonferroni multiple comparison post hoc test; * *p* ≤ 0.05. Note that fenofibrate significantly reduces the level of Cx43 in TGF-β_1_-treated HBFs.

In our recent report, we presented the key role of connexin 43 (Cx43), a protein that constitutes gap junctional channels between HBFs, in the regulation of the TGF-β_1_-induced FMT in HBF populations [23]. In accordance with these data, immunoenzymatic in-cell ELISA and Western blot analyses showed significantly increased Cx43 levels in the TGF-β_1_-treated HBFs, characterized by the highest sensitivity to TGF-β_1_/fenofibrate. Prompted by the attenuation of TGF-β_1_/Smad signaling and the decrease in α-SMA^+^ stress fiber formation in the fenofibrate-treated HBFs, we also examined the effect of fenofibrate on Cx43 levels in HBF populations. The combined TGF-β_1_/fenofibrate treatment attenuated Cx43 levels in comparison to the TGF-β_1_-treated cells (Figure 5A,B).

This phenomenon was confirmed by immunofluorescence studies and with cytofluorimetric analyses of HBFs (Figure 5C,D). These results clearly show the inhibitory effect of fenofibrate on Cx43 levels in the TGF-β_1_-treated bronchial fibroblasts, which corresponds to the attenuation of the TGF-β_1_/Smad2/3 signaling pathway and FMT efficiency.

## 3. Discussion

The majority of drugs currently available for bronchial asthma therapy do not significantly inhibit the fibrotic changes in the bronchial wall [29]. Only a few anti-inflammatory drugs used in the treatment of asthma (e.g., β_2_-adrenergic receptor agonists, glucocorticosteroids [29], methylxanthines [30], and statins [17]) were suggested to indirectly affect bronchial subepithelial fibrosis. However, numerous studies demonstrated anti-fibrotic effects of fenofibrate in models of hepatic [26,31], cardiac [32,33,34], renal [35], and lung [22] fibrosis, whereas the impact of fenofibrate on subepithelial fibrosis during asthma progression remains unaddressed. Our study fills this gap and shows, for the first time, that fenofibrate can efficiently interfere with the TGF-β_1_-induced myofibroblastic differentiation of bronchial fibroblasts derived from asthmatic patients.

Despite intensive research on bronchial remodeling in asthma, the mechanisms involved in the TGF-β_1_-induced FMT (the key event of subepithelial fibrosis in the asthmatic bronchial wall) are only partially understood. Heritable predilection of asthmatic HBFs may participate in this process. We recently showed that HBFs derived from asthmatic patients display a considerably higher susceptibility to TGF-β_1_–induced myofibroblastic differentiation than their non-asthmatic counterparts [14,23,36]. The induction of the FMT in HBF populations by the addition of exogenous TGF-β_1_ mimics the in vivo asthmatic process triggered by local secretion of TGF-β_1_ within the inflammatory milieu. TGF-β_1_ is one of the most important pro-fibrotic cytokines in vivo, and its expression increases in asthmatic airways [37]. Therefore, the experimental model, based on primary HBFs expanded directly from bronchial biopsies of different patients with diagnosed asthma (*n* = 10), is suitable for the analyses of the FMT background, as well as for pharmacokinetic studies. This model efficiently imitates the conditions and microenvironmental properties of asthmatic bronchial tissue. Using this model, we previously demonstrated that lovastatin and zaragozic acid A (inhibitors of the cholesterol synthesis pathway) reduce the efficiency of the TGF-β_1_-induced FMT in asthmatic HBF populations by lowering intracellular cholesterol levels [17]. Here, we show the inhibitory effect of fenofibrate on the TGF-β_1_-induced phenotypical transitions of HBFs. Notably, the inhibitory effects were reported in the presence of 10–25 μM fenofibrate, i.e., concentrations found in the sera of patients receiving therapeutic doses of fenofibrate [38,39,40]. Next, these effects were found in the absence of any visible cytotoxic effects [41,42]. This is an important finding, as HBF apoptosis or necrosis might result in improper responses of the immune system, leading to secondary inflammation [3]. Thus, dose-dependent inhibition of the TGF-β_1_-induced FMT in asthmatic HBF populations places this agent among many remarkable candidates for anti-fibrotic therapy in asthma.

Mechanistic studies demonstrated that fenofibrate attenuates the TGF-β_1_-induced FMT via the inhibition of the canonical Smad signaling pathway. This outcome is illustrated by significantly decreased Smad2/Smad3 phosphorylation levels and their attenuated nuclear translocation. Fenofibrate was already shown to inhibit the TGF-β_1_/Smad signaling axis in fibrotic processes in a model of diabetic nephropathy [43]. Previously, we showed the impact of statins [17] and methylxanthines [30] on the attenuation of the TGF-β_1_/Smad2 signaling axis in HBFs derived from asthma patients. Therefore, a fenofibrate-induced impairment of this signaling pathway may be associated with fenofibrate interference with cholesterol metabolism [31,44,45,46]. The possible mechanisms of this dependence may be based on its interference with the structure of membrane lipid rafts. These cholesterol-rich domains are disintegrated in response to cholesterol depletion, which conceivably can affect TGF-β_1_-dependent signaling [17,47]. However, fenofibrate displays pleiotropic activity; therefore, it can also affect the FMT of asthmatic HBFs in a manner independent of its lipid-lowering activity.

Furthermore, the upregulation of Cx43 levels observed in HBFs upon treatment of TGF-β_1_ was significantly and dose-dependently reduced by fenofibrate. Cx43 regulates cellular processes in two different ways: gap junctional intercellular coupling (GJIC)-dependent and GJIC-independent methods [48,49,50]. We previously showed GJIC-independent associations between TGF-β_1_/Smad2 signaling and TGF-β_1_-induced FMT and Cx43 function in HBFs [23]. The involvement of Cx43 in myofibroblast formation was also demonstrated in cardiac tissue [51]. These data indicate that fenofibrate interferes with TGF-β_1_/Smad2 signaling upstream of Cx43 involvement, which indirectly confirms the involvement of raft-dependent mechanism(s). However, we did not observe any considerable reduction in α-SMA^+^ upregulation in the TGF-β_1_/fenofibrate-treated HBFs. This apparent discrepancy can only be explained in terms of activation of fenofibrate-resistant signaling by TGF-β_1_ or by the involvement of other signaling pathways, leading to a pro-fibrotic response of these cells. Indeed, ERK1/2-dependent signaling, which was recently shown to participate in α-SMA^+^ regulation [23,28], is not attenuated by fenofibrate.

Rather than via the inhibition of α-SMA levels, fenofibrate may attenuate myofibroblast formation in HBF populations via inhibition of the α-SMA incorporation into stress fibers. Indeed, the impairment of the Smad2/3 signaling pathway was correlated with the inhibition of α-SMA^+^ incorporation into stress fibers. We previously showed that the Cx43-mediated GJIC regulates the formation of the α-SMA^+^ stress fibers. These mechanisms sustain α-SMA^+^ upregulation in the TGF-β_1_-treated HBFs; however, they do not allow for the integration of α-SMA^+^ into stress fibers. Chemical inhibition of GJIC does not affect the Cx43 and α-SMA levels, but significantly reduces the percentage of myofibroblasts [23]. Since fenofibrate-induced Cx43 downregulation inevitably impairs GJIC, we postulate the involvement of GJIC in this phenomenon. It is unclear whether the impairment of the α-SMA^+^ incorporation into stress fibers is the cause or effect of cytoskeletal rearrangements. However, similar rearrangements in the architecture of the actin cytoskeleton were demonstrated in fenofibrate-treated endothelial cells [42], which indicates the fenofibrate-specific nature of this effect. It is, thus, unambiguous that fenofibrate can prevent the excessive contractile activity of myofibroblasts in the asthmatic bronchial wall.

In summary, for the first time, we showed that fenofibrate directly affects the TGF-β_1_-induced FMT of human bronchial fibroblasts in vitro via the attenuation of the TGF-β_1_/Smad2/3 axis and a concomitant block of α-SMA incorporation into stress fibers via connexin 43 function. Because the FMT is crucial for the asthmatic process, the application of fenofibrate should potentially be considered in the chemoprevention of subepithelial fibrosis in asthma. A clinical and therapeutic potential of fenofibrate may also be expanded for the future development of new viable therapeutic approaches for asthma medication.

## 4. Materials and Methods

### 4.1. HBF In Vitro Cultures

The primary human bronchial fibroblasts (HBFs) were established as described previously [52] from bronchial biopsy explants derived from 10 patients with asthma severity 3–4 (Global Initiative for Asthma (GINA) classification). Briefly, all individuals were treated in the Department of Medicine of the Jagiellonian University Medical College and remained in stable clinical conditions. The study group consisted of five females and five males aged 36.7 ± 11.1 years. The patients were characterized by the reduced value of the forced expiratory volume in one second (the average FEV_1_%: 67.3 ± 22.5). The mean duration of asthma in the experimental group was 9.9 ± 6.1 years. The study was approved by the Jagiellonian University Ethics Committee (Decision No. 122.6120.16.2016; 28 January 2016) and all the patients provided informed consent to participate. Phenotypes of the established primary HBF cultures were verified by immunofluorescence staining of α-smooth muscle actin (α-SMA), vimentin, and desmin [53]. All cells expressed vimentin-positive staining, whereas the expression of desmin was not observed. In the HBF populations, α-SMA-positive staining (α-SMA^+^) was observed in ca. 5% of cells. HBFs were cultured in a complete medium (Dulbecco’s modified Eagle medium; Sigma-Aldrich, St. Louis, MO, USA), supplemented with 10% fetal bovine serum (FBS; Gibco^TM^, Thermo Fisher Scientific, Waltham, MA, USA), and a penicillin/streptomycin cocktail (P4333; Sigma-Aldrich, St. Louis, MO, USA) as described previously [23,30]. For each experiment (unless otherwise noted), the cells were seeded at a density of 5000 cells/cm^2^ and cultured in complete medium for 24 h, followed by 24 h in serum-free medium containing 0.1% bovine serum albumin (BSA; Sigma-Aldrich, St. Louis, MO, USA). When indicated, human recombinant TGF-β_1_ (5 ng/mL, BD Bioscience) and/or fenofibrate (1–50 µM, stock: 8 mM in dimethyl sulfoxide (DMSO); Sigma-Aldrich, St. Louis, MO, USA) were administered.

### 4.2. Viability and Proliferation Tests

Cell viability was determined after one, two, and seven days of incubation with fenofibrate (1–50 µM) by the fluorescein diacetate (FDA)/ethidium bromide (EtBr) test [17] using a Leica DM IRE2 microscope, and the results are expressed as the percentage of the FDA^+^/ EtBr^−^ cells.

For the proliferation tests, the cells were cultured in complete medium for 24 h and then in a serum-free medium with or without fenofibrate (0–50 µM) for varied times, from one to seven days. Then, the cells were fixed with 3.7% formaldehyde/PBS, and the crystal violet assay was performed [30]. The results are shown as absorbance values (540 nm).

### 4.3. Movement of Individual Cells—Time-Lapse Monitoring

The cells were seeded into 12-well plates. After that, fenofibrate (25 µM) was added for 48 h to the proper wells. Time-lapse imaging was performed with a fully motorized Leica DMI6000B microscope equipped with a monochrome digital DFC360FX CCD camera (all by Leica Microsystems, Wetzlar, Germany). A temperature of 37 °C and a 5% CO_2_ concentration were maintained with an environmental control system. Images were captured with integrated modulation contrast (IMC) every 15 min for 13 h. The obtained image series was analyzed manually with the Hiro 1.0.0.4 software. Trajectories for 50 individual cell centroids were constructed and presented as circular diagrams, with the starting points situated at the plot center. The parameters describing the motility of the cells, the average speed of cell movement (ASCM; µm/h), and the average rate of cell displacement (ARCD; µm/h), were calculated as described elsewhere [41,54,55].

### 4.4. Cholesterol Content Assay

For the experiments, the cells were grown in complete medium for seven days (at a density of 3250 cells/cm^2^). Isolation of intracellular cholesterol was carried out in duplicate using a 750-µL hexane/isopropanol mixture (3:2) per well for 30 min with shaking. The supernatants were collected in glass bottles and evaporated under a gentle stream of nitrogen. Cholesterol levels were determined with the use of an Amplex^TM^ Red Cholesterol Assay Kit (Invitrogen^TM^, Thermo Fisher Scientific, Waltham, MA, USA) according to the manufacturer’s protocol, and measured using an Infinite M200PRO microplate reader (Tecan; excitation of fluorescence: 540 nm; emission: 590 nm). The results were converted relative to the cholesterol curve and are shown as the concentration of cholesterol (µg/mL).

### 4.5. Immunofluorescence Staining

For the immunocytofluorescence analyses, HBFs were cultured in 24-well plates (µPlate 24 well; ibidi) in control or experimental conditions for a varied period (1 h for p-Smad2; seven days for α-SMA, Cx43, and vinculin analyses). Afterward, the cells were fixed with 3.7% formaldehyde/PBS, permeabilized with 0.1% Triton X-100/PBS, blocked with 1% BSA/PBS (Sigma-Aldrich), and incubated with primary antibodies: mouse monoclonal IgG against α-SMA (A2547, clone 1A4, 1:400); rabbit polyclonal IgG against connexin 43 (C6219, 1:200), rabbit polyclonal IgG against p(Ser467)-Smad2 (SAB4300251, 1:100), rabbit polyclonal IgG against Smad2 (SAB4501808, 1:100), and mouse monoclonal IgG against vinculin (V9131, 1:1000), all from Sigma-Aldrich, St. Louis, MO, USA, and appropriate secondary antibodies were applied: AlexaFluor488-conjugated goat anti-mouse IgG and AlexaFluor546-conjugated goat anti-rabbit IgG (all: 1:500, Life Technologies, Thermo Fisher Scientific, Waltham, MA, USA). Some were counterstained with Hoechst 33342 (1 µg/mL, Sigma-Aldrich, St. Louis, MO, USA) and phalloidin conjugated with AlexaFluor546 (Life Technologies, Thermo Fisher Scientific, Waltham, MA, USA). Images were acquired using a Leica DMI6000B inverted microscope (Leica Microsystems, Wetzlar, Germany) equipped with the LAS-X software for image processing. The FMT efficiency was counted as the percentage of cells with α-SMA-positive microfilaments in the HBF populations. Similarly, activity of the Smad signaling pathway was determined by the percentage of cells expressing a strong signal in the nuclear area from p-Smad2 staining. Quantitative analysis of actin cytoskeletons was performed with Fiji ImageJ software, version 1.51s [56]. Plot profiles perpendicular to bundles of stress fibers were created, and the signal for particular spikes was analyzed. The averaged signal for at least 50 stress fibers was compared between the conditions. Cytofluorimetric analyses of Cx43 levels were performed using the same excitation/exposure settings as described previously [23,57].

### 4.6. Focal Adhesion Imaging

Visualization of focal adhesions (FAs) was conducted with the same fluorescence microscope, equipped with a high-numerical aperture objective of 100× magnification and total internal reflection fluorescence (TIRF) module. TIRF microscopy was used to selectively visualize vinculin near the plasma membrane. To obtain quantitative data, HBFs were transfected with Lipofectamine 2000 transfection reagent and mEmerald-Vinculin-23 plasmid, which was a gift from Michael Davidson (Addgene plasmid #54302). Then, 24 h after transfection, the cells were incubated with TGF-β_1_ and visualized five days later. Captured images were analyzed automatically with the Fiji ImageJ software, version 1.51s, and a macro based on the algorithm published previously [58] with minor modifications. The analysis provided data about the area and length of particular focal contacts. The results are presented as a fraction of the overall FA area covered by FAs of particular types.

### 4.7. Protein Extraction and Western Blotting

HBF cultures were lysed as described previously [23,30], and the protein content in the supernatant was determined using the Bradford method. Protein samples (30 µg/lane) were electrophoresed on 10% SDS-polyacrylamide gels and transferred to polyvinylidene difluoride (PVDF) membranes (Bio Rad, Hercules, CA, USA) as described previously [23,57]. Next, after blocking, the membranes were incubated overnight at 4 °C with primary antibodies: rabbit polyclonal IgG against p(Ser467)-Smad2 (1:500), rabbit polyclonal IgG against Cx43 (1:2000), mouse monoclonal IgG against α-SMA (1:2000), mouse monoclonal IgG against vinculin (1:1000), mouse monoclonal IgG against β-tubulin (1:1000), and mouse monoclonal IgG against glyceraldehyde-3-phosphate dehydrogenase GAPDH (1:3000), all from Sigma-Aldrich, St. Louis, MO, USA; rabbit monoclonal IgG against Smad2 (D43B4), rabbit monoclonal IgG against p(Ser465/467)-Smad2 (138D4), rabbit monoclonal IgG against Smad3 (C67H9), rabbit monoclonal IgG against p(Ser423/425)-Smad3 (C25A9), rabbit polyclonal IgG against Focal adhesion kinase (FAK), rabbit polyclonal IgG against p(Thr202/Tyr204)-Erk1/2, and rabbit polyclonal IgG against Erk1/2 (all: 1:500; Cell Signaling Technology^®^ Danvers, MA, USA).

After triple-washing with TBST (10 mM Tris–HCl, 150 mM NaCl, 0.05% Tween-20), horseradish peroxidase (HRP)-conjugated anti-mouse or anti-rabbit IgG (all: 1:3000; Life Technologies) diluted in 2.5% skim milk/TBST were added for 1 h. For band detection, Luminata Crescendo Western HRP Substrate (Merck Millipore, Burlington, MA, USA) and a chemiluminescence imaging system (MicroChemi; DNR Bio-Imaging Systems, Jerusalem, Israel) were used. Band intensities were quantified using the Fiji ImageJ software, version 1.51s.

### 4.8. Measurement of Protein Levels Using In-Cell ELISA

The cells were seeded into 96-well plates and treated with a fresh serum-free medium containing recombinant TGF-β_1_ (5 ng/mL) alone or in combination with fenofibrate (1, 10, or 25 µM) for seven days. The in-cell ELISA protocol [30] was performed using antibodies from a Focal Adhesion Protein Antibody Sampler Kit (13430T; Cell Signaling Technology^®^), rabbit polyclonal anti-fibronectin (Sigma-Aldrich, St. Louis, MO, USA), or those against α-SMA and Cx43 (1:2000 in 1% BSA/PBS, Sigma-Aldrich, St. Louis, MO, USA). The results are shown as the absorbance value (450 nm; Microplate Reader, Thermo Scientific, Multiskan FC) corresponding to the relative amount of protein level.

### 4.9. Statistics

Data are presented as the mean ± standard error of the mean (SEM) from at least three independent experiments. Comparisons between groups were performed by one-way or two-way analysis of variance (ANOVA) followed by the post hoc Bonferroni multiple comparison test. Differences were considered statistically significant at a *p*-value < 0.05 (*) or <0.001 (***).
